# Using intersectionality to study gender and antimicrobial resistance in low- and middle-income countries

**DOI:** 10.1093/heapol/czad054

**Published:** 2023-07-27

**Authors:** Juliette M C Gautron, Giada Tu Thanh, Violet Barasa, Giovanna Voltolina

**Affiliations:** Department of Social Anthropology, University of Cambridge, Free School Lane, Cambridge, CB2 3RF, United Kingdom; Independent Consultant, Gran de Gracia, Barcelona 08012, Spain; Institute of Development Studies, University of Sussex, Library Road, Brighton & Hove, BN1 9RE, United Kingdom; Itad, Preece House, Davigdor Road, Brighton & Hove, BN3 1RE, United Kingdom

**Keywords:** Social epidemiology, health inequalities, social determinants, gender, health system research, rational drug use, access, health care, equity, developing countries

## Abstract

Different sexes and genders experience differentiated risks of acquiring infections, including drug-resistant infections, and of becoming ill. Different genders also have different health-seeking behaviours that shape their likelihood of having access to and appropriately using and administering antimicrobials. Consequently, they are distinctly affected by antimicrobial resistance (AMR). As such, it is crucial to incorporate perspectives on sex and gender in the study of both AMR and antimicrobial use in order to present a full picture of AMR’s drivers and impact. An intersectional approach to understanding gender and AMR can display how gender and other components ‘intersect’ to shape the experiences of individuals and groups affected by AMR. However, there are insufficient data on the burden of AMR disaggregated by gender and other socio-economic characteristics, and where available, it is fragmented. For example, to date, the best estimate of the global burden of bacterial AMR published in *The Lancet* does not consider gender or other social stratifiers in its analysis. To address this evidence gap, we undertook a scoping review to examine how sex and gender compounded by other axes of marginalization influence one’s vulnerability and exposure to AMR as well as one’s access to and use of antimicrobials. We undertook a gendered analysis of AMR, using intersectionality as a concept to help us understand the multiple and overlapping ways in which different people experience exposure vulnerability to AMR. This approach is crucial in informing a more nuanced view of the burden and drivers of AMR. The intersectional gender lens should be taken into account in AMR surveillance, antimicrobial stewardship, infection prevention and control and public and professional awareness efforts, both donor and government funded, as well as national and international policies and programmes tackling AMR such as through national action plans.

Key messages

- Different sexes and genders experience differentiated risks of acquiring bacterial infections, including drug-resistant infections, and of becoming ill. Different genders also have different health-seeking behaviours. Consequently, they are distinctly affected by antimicrobial resistance (AMR).
- Disaggregated data on the burden of AMR by gender and other characteristics are lacking and, where available, fragmented. Hence, perspectives on sex, gender and other socio-economic characteristics need to be incorporated into the study of both AMR and antimicrobial use (AMU).
- An intersectionality approach helps highlight how gender and other axes of marginalization that are common in low- and middle-income countries contribute to the exacerbated vulnerability to AMR.
- A more nuanced view of the burden of AMR on specific categories of the population as well as its drivers should inform not only any AMR interventions globally, including for national action plans, but also AMR surveillance, AMU monitoring, infection prevention and control and public and professional awareness measures, both donor and government funded.

## Introduction

It is estimated that in 2019, 4.95 million deaths were associated with bacterial antimicrobial resistance (AMR), the highest death rate of which is in western and sub-Saharan Africa, with 27.3 deaths per 100 000 (Murray *et al.*, 2022). It is estimated that ‘unless action is taken, the burden of deaths from AMR could balloon to 10 million lives each year by 2050’ (The Review on Antimicrobial Resistance, 2016). AMR is a collective term that describes the ability of microorganisms, including bacteria, fungi, viruses and parasites, to change over time and no longer respond to previously effective antimicrobials. AMR makes existing antimicrobials increasingly less effective, thereby threatening the prevention and treatment of an ever-expanding range of infections and posing a serious threat to global public health that requires urgent and concerted action (WHO, 2022).

The drivers of AMR are complex and multifaceted and differ from one place to another. In low- and middle-income countries (LMICs), evidence suggests that the main drivers of AMR include the misuse and overuse of antimicrobials; lack of access to clean water, sanitation and hygiene (WASH); limited resources for infection prevention and control (IPC) in healthcare facilities and farms; limited access to quality, affordable medicines, vaccines and diagnostics; lack of awareness and knowledge and lack of enforcement of legislation (WHO, 2021a). The most common transmission routes for drug-resistant organisms (DROs) include human-to-human transmission, contact with contaminated medical equipment, medical waste, human–animal interaction, environmental exposure and contaminated food (Godijk *et al.*, 2022).

Nowhere is the burden of AMR greater than in low-resource settings where weak health systems, including a lack of health information systems and limited resources, compromise AMR surveillance (Iskandar *et al.*, 2021). Nevertheless, we note that the economic burden of AMR is not confined to LMICs. In Europe alone, the annual cost of AMR is estimated at €9 billion (Dagostar, 2019). Based on scenarios of rising drug resistance for six pathogens, it is estimated that, by 2050, the burden of deaths from AMR would rise to a cumulative cost to global economic output of US$100 trillion each year (The Review on Antimicrobial Resistance, 2016). These losses are expected to accumulate globally to US$85 trillion in gross domestic product and US$23 trillion in global trade (present value) by 2050 (Ahmed *et al.*, 2018). This increase in expenditure and mortality and decrease in health and fitness will be reflected in a multi-country and multisector reduction in labour productivity, increasing healthcare costs, disability and the effects of poverty.

The general economic impacts of the threats of AMR on global health are well understood. However, the impacts of AMR go beyond the global economy and have dire consequences and more pervasive social impacts, which can disproportionately affect certain groups compared with others. While an economic approach may be informative, it lacks the ability to put a ‘human face’ to the problem (WHO, 2018). Long and chronic disease progression, commonly associated with antibiotic-resistant pathogens, can have a severe impact on an individual’s quality of life, physical fitness and mortality (Abrahamian *et al.*, 2011; Koukoubani *et al.*, 2021). If nothing is done to stop or slow down the spread of AMR, these negative outcomes could become more pronounced. As the fastest emergence of highly resistant pathogens occurs among older patients, due to the prevalence of infectious diseases in this population (Dabota Buowari, 2017), life expectancy will be shortened unless preventive strategies coupled with new drug developments are established soon (Yoshikawa, 2002).

The impacts of AMR are however not the same for everyone, even for people with shared socio-economic characteristics. Different sexes and genders experience differentiated risks of acquiring bacterial infections, including drug-resistant infections (DRIs), and of becoming ill. Different genders also have different health-seeking behaviours (HSBs) and differentiated access to health care and education that shape their likelihood of appropriately using and administering antimicrobials (Jones *et al.*, 2022). Therefore, to understand these differential impacts, pathways and key drivers of AMR, it is important to unpack the burden of AMR, not only from an economic, disease or epidemiological perspective but also from a social and anthropological one.

Nevertheless, data on the burden of AMR disaggregated by sex, gender or other social variables (such as age, ethnicity, disability, geographical location, occupation, sexual orientation and socio-economic status) are still missing. For example, to date, the best estimate of the global AMR burden (Murray *et al.*, 2022) does not consider gender or other social stratifiers in its data analysis. The literature explicitly addressing the linkage between sex, gender and AMR is scarce even though such an approach would provide a better understanding of how AMR impacts different segments of the population and how these impacts are experienced by different groups, which would assist the development of targeted strategies to tackle AMR nationally and globally. National action plans on AMR often fail to incorporate gender and other socio-economic characteristics, partly due to the lack of research on the sex- and gender-disaggregated impacts of AMR. The World Health Organization (WHO) has recognized the need to ‘take the first step towards better considering gender and equity issues in [countries’] efforts to tackle AMR, to inform the implementation of strategies in national action plans on AMR, and contribute to improved reach and effectiveness of AMR efforts in the longer term’ (WHO, 2018, p. 1).

This paper presents and discusses the result of a scoping review conducted to explore the body of literature that bridges sex and gender, on the one hand, and drivers and impact of AMR, on the other hand. In doing so, this paper makes the case for the need to incorporate intersectional gender perspectives into the study of both AMR and antimicrobial use (AMU). Sex- and gender-disaggregated data and an intersectional analysis of this can provide development actors and policymakers with a more nuanced view of the burden of AMR on specific categories of the population as well as its complex drivers to better tailor strategies and programmes aimed at reducing its emergence and spread while maximizing equity and social justice mandates. This would in turn contribute to achieving better and more equitable outcomes in reducing the emergence and spread of AMR globally. It is also central to the attainment of many sustainable development goals (WHO, 2018).

In this paper, we understand ‘sex’ as the biological and chromosomal reality of one’s body, which categorizes male, female and intersex people (Blackless *et al.*, 2000), while ‘gender’ is a psychosocial construct, which is not located in the body and which categorizes people as men, women and non-binary. Gender is socially constructed and relates to ‘the psychological, behavioural, and social characteristics [often] attributed to a particular sex, as perceived by a person or the society they live in’ (Hakeem, 2018, p. 11). It includes ‘norms, behaviours and roles’ (WHO, 2022) associated with women, men, girls, boys or people with non-binary identities and shapes their interactions with each other. ‘Gender varies from society to society and can change over time’ (WHO, 2022). Sex and gender are often intertwined, with gender often being a socialized aspect of sex. While our understanding of gender is non-binary, the findings presented in this paper are predominantly binary, with a focus on cisgender men and women.

While gender plays a key role in shaping the health experience of an individual, whether through one’s exposure to pathogens due to gendered activities or through one’s gendered HSB, an approach with a single gender axis has numerous limitations (Hankivsky, 2012). It is the intersection and interaction of gender and the various social, economic and political barriers ‘that produce varying levels of health inequalities and exclusion for specific individuals or groups in a particular setting. These intersections are often underappreciated in health research’ (Barasa and Virhia, 2022, p. 2). Gender intersects with other social stratifiers^1^ such as occupation, income, age, geographic location, marital status, education and disability in mutable and dynamic ways to exacerbate inequalities in access to information as well as quality health care by specific people in specific places (McCollum *et al.*, 2019).

This paper starts by outlining the methodology and limitations of our approach. We then present findings on sex, gender, exposure and vulnerability to AMR through a focus on WASH, frontline health workers, animal husbandry, food handling and preparation, malnutrition and sexual behaviours. We proceed to unpack the link between gender and antimicrobial prescription, use and administration and then subsequently discuss the implications of our findings for the study of AMR. We conclude the paper by presenting conclusions and areas for further research and policy.

## Methods

This scoping review was focused on answering the following two research questions:

1. How do sex and gender, compounded by other social stratifiers, influence vulnerability and exposure to AMR?
2. How does gender influence HSBs related to AMR such as antimicrobial access, use and administration to others?

We used two main papers (WHO, 2018; ReACT, 2020) as the starting points of our enquiry because of their novelty and pioneering stance in bridging the gap between AMR and gender. We followed with backward citation tracing^1^ to locate additional papers and broaden the scope of our review and supplemented these results with a standard keyword-based search.

We conducted the keyword-based search on the following platforms: the National Centre for Biotechnology Information, BioMed Central, Science Direct and Google Scholar. Databases screened for the United Nations (UN) agencies’ reports included the UN iLibrary, the WHO Online Library and the WHO Institutional Repository for Information Sharing. The main search terms used included a combination of AMR-related terms, gender-related terms and topic-specific terms (AppendixTable A1). The last search was conducted in May 2023.

This search retrieved peer-reviewed literature, including primary research, systematic, scoping and literature reviews, commentaries, author correspondence and opinion papers. These papers encompassed a variety of methods and approaches, from experimental and quasi-experimental designs and observational studies to qualitative studies using ethnography, feminist research, action research or grounded theory. This wide scope was justified by the limited literature that links AMR and gender and our aim to scope out the available literature across all fields.

This search also included grey literature, which included reports published by UN bodies or agencies.

The titles and abstracts (or table of contents and introductions for reports) of retrieved texts were initially screened according to their relevance to the two main research questions outlined earlier and subsequently screened according to our inclusion criteria (listed in Table 1). In Table 2, we present the distribution of our shortlisted papers by decade.

**Table 1.** Criteria for inclusion in this study

Inclusion criteria
1. Papers written in English
2. Papers published after the year 2000
3. Papers drawing a direct link between either AMR and gender or gender and infection
4. Papers that focused on gender differences in LMICs
5. Papers available online
6. Papers whose full text could be accessed

**Table 2.** Distribution of shortlisted papers, by decade

Decade	Publications
2000–09	14 (12%)
2010–19	62 (54%)
2020–23	39 (34%)
Total	115 (100%)

The first author developed a data-charting form in collaboration with the second author and used it for data extraction. This process was followed by a thematic analysis, through which the main themes for this paper were identified, as presented in Table 3. We further categorized these umbrella areas under two main headings; they are sex and gender: vulnerability and exposure to AMR and gender and antimicrobial access, use and administration.

**Table 3.** Number of included studies, by type and thematic area

Thematic areas	Peer-reviewed literature	Technical reports by UN agencies
Sex and gender: vulnerability and exposure to AMR	86	12
*WASH*	*32*	*5*
*Frontline health workers*	*6*	*0*
*Animal husbandry*	*5*	*1*
*Food handling and preparation*	*6*	*1*
*Malnutrition*	*7*	*1*
*Sexual behaviours*	*30*	*4*
Gender and antibiotic access, use and administration	15	2
**Total (115)**	**101**	**14**

**Figure 1.**
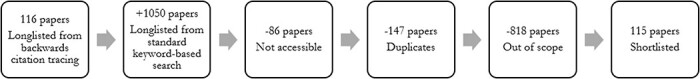
Identifying publications for review

We then used a standard keyword-based search to supplement the literature within each thematic area. Finally, we summarized the types of setting, population and study for each paper included, along with its broad findings. The selection process is depicted in Figure 1.

Evidence from the reviewed literature has been analysed using an intersectional gender lens, which provides a framework to acknowledge how different genders, and different groups in society, may be at risk of or impacted differently by AMR and by the efforts to address it (WHO, 2018). Intersectionality (Crenshaw, 1989) is a concept that highlights how overlapping markers of identity can result in systemic marginalization. These markers may be social, cultural, ethnic, political, sexual or economic, among others. An intersectional gender lens is a concept increasingly used in health research but underappreciated in studies on gender and AMR (McCollum *et al.*, 2019). This approach should help highlight how gender compounded by other axes of marginalization that are common in low-resource settings contributes to exacerbated vulnerability and exposure to as well as drivers of AMR. Intersectionality as a concept and an intersectional gender lens was used as frameworks and theories that underpinned our research and analysis.

### Limitations

This paper provides social science perspectives on sex and gender determinants of exposure to DROs and vulnerability to DRIs and AMU. In doing so, it explores various social and cultural factors that impact disease transmission, vulnerability to infections with the potential of being or becoming drug resistant and HSBs in low-resource settings. One of the limitations of doing this is that the paper cannot empirically establish the causal relationship between these factors and AMR, and therefore, the relationship is only an implied one.

Due to a lack of relevant data, we recognize that our findings are limited to the available literature on AMR and gender that focuses on LMICs, meaning that other genders are not represented. The literature on gender in the health sector and more broadly the development sector often adopts a binary definition of gender. There is limited literature available in the health sector on individuals who do not ascribe to the binary definitions of gender, particularly within LMICs. The literature with a medical and epidemiological focus on non-binary individuals in LMICs and their experience of infection or access to health care was difficult to locate and may require further primary research. Due to these constraints, this study focuses on gender through the lens of ‘men’ and ‘women’ and focuses on the ‘female’ and ‘male’ sex categories.

Generalizability of the results of our scoping review is limited by the fact that the majority of the available literature we analysed is context specific and examples are tied to specific contexts within individual countries, which limits the ability to generalize the assumptions and links made in each subtheme explored in this paper.

Finally, we acknowledge that while this scoping review provides a starting point to the intersectional study of AMR, it does not aim to list all the potential vulnerabilities, exposure risks and drivers of AMR, for all population categories.

## Results

Based on the inclusion criteria highlighted earlier, 1166 papers were found, 115 of which were retained as part of the review.

### Sex and gender: vulnerability and exposure to AMR

Sex and gender interact to influence differences in infections. Biological sex tends to influence ‘susceptibility to infection, pathophysiology, immune responses, clinical presentation, disease severity, and response to treatment and vaccination’ (Dias *et al.*, 2022). On the other hand, gender roles and social norms can influence risk factors and exposure to infection and determine HSBs (Dias *et al.*, 2022).

Occupations that may influence the likelihood of exposure to DROs may include health care (Clements *et al.*, 2008; O’Donnell *et al.*, 2010; Tacconelli *et al.*, 2014; Wang *et al.*, 2017), animal husbandry and slaughter (Adelaide *et al.*, 2008; Kawaguchi *et al.*, 2008; Skufka and Arima, 2012; Hrynick *et al.*, 2019; Barasa and Virhia, 2022) or sex work (Jeal and Salisbury, 2004; Zarakolu *et al.*, 2006; Kenyon and Schwartz, 2018). Such occupations have a gendered aspect to consider, which increases the likelihood of acquiring a DRO (and therefore of developing a DRI), for both men and women.

Gender determines the division of labour in many contexts including LMICs and high-income countries (HICs). Limited access to safe water particularly impacts women and young girls as they generally fetch water for the household (UNDESA, 2010) and may be more exposed to contaminated runoff or contaminated surface water (WHO, FAO & OIE, 2020). Limited access to sanitation and hygiene further affects females due to their physiological characteristics (Muhammed, 2014; Das *et al.*, 2015; Torondel *et al.*, 2018; Kumar, 2019).

Intimate relationships and sexual behaviours also highlight the intersectional nature of exposure and vulnerability to AMR, whereby biological characteristics of one’s sex intersect with one’s sexual orientation, gendered roles within a relationship, sexual violence and sex work (Vandepitte *et al.*, 2014; Tsuboi *et al.*, 2021; Kaggwa *et al.*, 2022; Yakobi and Pooe, 2022).

The rest of this section highlights the various ways in which sex, gender and their intersecting variables influence exposure to DROs and vulnerability to DRIs.

#### WASH

One of the main drivers of AMR is the lack of access to clean WASH for both humans and animals (WHO, 2021a). Lack of access to clean water is the primary environmental AMR reservoir and dissemination pathway (Kaiser *et al.*, 2022). Globally, at least 2 billion people use a drinking water source contaminated with faeces (UNICEF & WHO, 2019a). Fourteen per cent of people carry *Escherichia coli* in their faeces that produce extended-spectrum beta-lactamase enzymes that provide resistance to antibiotics such as penicillins, cephalosporins, cephamycins and to some extent carbapenems (Karanika *et al.*, 2016). In addition, up to 80% of the antimicrobial dose administered can be excreted as the active compound depending on the class of antimicrobial usage, and wastewater treatment is often insufficient or not possible (Larsson, 2014). Antimicrobials in water downstream of some antimicrobial manufacturing sites have been found at concentrations higher than in the blood of patients taking medicines (Larsson, 2014). A total of 785 million people lack even a basic drinking water service, including 144 million people who are dependent on surface water (WHO, FAO & OIE, 2020). In LMICs, women and girls overwhelmingly bear the burden of water provision. This is particularly true in rural areas in sub-Saharan Africa and Asia where water infrastructure is lacking (UNDESA, 2010). Women and girls may therefore be at an increased risk of exposure to AMR-contaminated runoff. Entire households, especially children, may be susceptible to waterborne diseases due to the consumption of contaminated water as household-level contaminants are common in rural settings in LMICs. In water-stressed settings, multiple studies show that household water handling and storage practices contribute to the prevalence of *E. coli* in children (Parker *et al.*, 2010; Agensi *et al.*, 2019), which is the leading pathogen contributing to AMR-related deaths (Murray *et al.*, 2022).

In low-resource settings with inadequate sanitation, exposure to faecal matter increases the exposure to antibiotic-resistant pathogens. A study in rural Zimbabwe showed that children were extremely exposed to high levels of *E. coli* in their play environments, in compounds or outdoors. Their ingestion of soil and chicken faeces put them at particular risk of being exposed to *E. coli* (Ngure *et al.*, 2013). Livestock-keeping communities, as further discussed in the later section on animal husbandry, similarly face heightened risks of contracting infectious diseases like leptospirosis (Skufka and Arima, 2012, p. 38), which spreads by contact with water contaminated with the urine of infected animals. For example, children are more likely to walk barefoot in contaminated soil and groundwater after rainfall, which is a major risk factor for infection in developing countries, due to frequent exposure to animal waste (Kawaguchi *et al.*, 2008, p. 960).

In addition to the exposure to drug-resistant pathogens, children of all genders, parents and particularly women are at further risk of infections and acquiring a DRO, due to their caregiving role (Evans, 2010; Sharma *et al.*, 2016). As part of caregiving responsibilities, exposure to faecal matter through the mismanagement of child faeces through the use of unsafe tools may increase exposure to pathogens carrying resistance such as *E. coli* for the primary caregiver (Bauza *et al.*, 2020). The risk is heightened for women in low-resource settings who have low levels of education and low access to WASH facilities, such as studies demonstrated in Nigeria (Aluko, et al., 2017), India (Bauza *et al.*, 2020) and Bangladesh (Islam *et al.*, 2018).

The risk of developing urinary tract infections (UTIs), which have a high drug resistance potential, is increased in low-resource settings whereby WASH facilities are not readily available (Kawade *et al.*, 2019; Nabwera *et al.*, 2021).

Menstruation is a key time during which menstruating people are at a higher risk of contracting UTIs that can be drug resistant. The lack of menstrual hygiene management leads to an increased risk of urogenital infections (Das *et al.*, 2015; Torondel *et al.*, 2018), which are particularly prevalent in areas with poor menstrual hygiene occasioned by limited access to sanitary products and clean water and where menstruation is taboo. For example, in India, menstruating individuals tend to use a loincloth made from old saris or bleed freely due to a lack of sanitary pads (Kumari *et al.*, 2021). Repeated use of a dirty cloth and improper drying before reuse it results in the presence of microorganisms, which may result in the spread of vaginal infections (Kumari *et al.*, 2021). Menstrual seclusion practices further enhance poor access to adequate sanitation and menstrual hygiene products. For example, in some rural areas of Nepal, menstruating individuals comply with isolating practices during menstruation, whereby they leave the house and stay in menstrual huts, with very basic WASH facilities (Nawal, 2020). During that time, they are considered impure and are banned from accessing common household facilities. These practices tend to put women and girls in low-resource settings at risk of AMR-related infections including UTIs and reproductive tract infections (RTIs). These examples demonstrate how gender interacts with biological and socio-economic factors to exacerbate women’s and girls’ vulnerability to potential DROs.

Furthermore, the above-mentioned gendered exposure to AMR and vulnerability to drug-resistant UTIs intersect through the biological reality of the female body, increasing the risk factor of developing a UTI for women. Females are at a higher risk than males of contracting a UTI due to the physical characteristics of their sex, such as having a shorter urethra, meaning that bacteria are more likely to infect the bladder (Muhammed, 2014; Kumar, 2019).

UTIs are also the most common infection during pregnancy due to hormonal fluctuations (Ghouri and Hollywood, 2020), thus increasing females’ risk of developing a drug-resistant UTI. The UTI prevalence is higher in females than in males overall, especially at younger ages and for pregnant individuals (Rowe and Juthani-Mehta, 2013; Yeta *et al.*, 2021). Unclean births may also heighten the risk of infections for pregnant individuals and their babies, accounting for 26% of neonatal deaths and 11% of maternal mortality, which adds up to >1 million deaths each year (Say *et al.*, 2014; WHO, FAO & OIE, 2020). Studies have shown a high prevalence of drug-resistant UTIs particularly among pregnant individuals, such as in Zambia (Yeta *et al.*, 2021) and in Ethiopia (Chelkeba *et al.*, 2022). A study comparing the quality of life for females with antibiotic-susceptible and antibiotic-resistant UTIs reported that females with resistant strains missed three times as many hours of normal daily activities, missed more work hours and spent more time in bed than patients with susceptible strains, by up to 6 weeks, with obvious socio-economic impacts (Abrahamian *et al.*, 2011). Patients with multidrug resistant (MDR) uropathogens also tend to have prolonged hospital stays (Mohamed *et al.*, 2022). This highlights the impact AMR can have on individuals’ quality of life and their socio-economic situation.

Elderly patients tend to be correlated with increased drug-resistant UTIs (Lin *et al.*, 2022; Mohamed *et al.*, 2022). In the elderly, however, the UTI prevalence in males increases and can be higher than in females (Akoachere *et al.*, 2012), a difference that may be attributed to the likelihood of older males spending more time in hospital settings than older females (Horcajada *et al.*, 2013). Approximately 25% of patients who are hospitalized have an indwelling urinary tract catheter placed at some time during their hospital stay, with high levels of catheter-associated UTIs caused by some highly resistant pathogens (Ko *et al.*, 2008). Fifteen per cent of patients in LMICs acquire one or more infections during a hospital stay (Allegranzi *et al.*, 2011). Low levels of IPC and access to WASH facilities in low-resource settings increase the risk of infections and DRIs spreading within healthcare facilities. For example, one in five healthcare facilities has no sanitation service, meaning that >1.5 billion people are going to healthcare facilities without toilets (UNICEF & WHO, 2019b). Forty-two per cent of healthcare facilities lack hand hygiene facilities at the point of care and 40% do not have systems to segregate waste (UNICEF & WHO, 2019b). These examples highlight how vulnerability and exposure to pathogens and hospital-acquired infections may affect genders differently at different life stages.

As shown in this section, access to WASH is key to ensuring the health of women and reducing the prevalence of UTIs (Nabwera *et al.*, 2021). The risk of infections, UTIs and drug-resistant UTIs is accrued at the intersection of gendered practices of menstrual seclusion, unsafe menstrual hygiene management and sex-based differences such as the shorter female urethra. All these aspects are compounded by living in low-resource settings with inadequate access to WASH facilities. In addition, limited WASH infrastructures within healthcare facilities further increase the risk of infection in not only patients but also frontline health workers, as further developed later. It is possible to highlight here the various layers of risk, exposure or vulnerability faced by women in the context of limited access to WASH facilities through the use of an intersectional gender lens.

#### Frontline health workers

As discussed earlier, low levels of IPC and limited WASH facilities may drive the spread of pathogens within healthcare centres. Healthcare facilities often have high AMU and hold a high concentration of sick people, increasing the spread of resistant strains of bacteria (Tacconelli *et al.*, 2014). High antibiotic use, insufficient sanitation, poor hygiene practices and improper cleaning may facilitate the spread of resistant strains within clinics or facilities (Tacconelli *et al.*, 2014). Furthermore, overcrowding, understaffing and limited numbers of isolation rooms can further increase the spread of resistant bacteria (Clements *et al.*, 2008).

Healthcare workers have a particularly high risk of contracting infections such as tubercolosis (TB), including drug-resistant TB: a South African study highlights that extensively drug-resistant-TB and MDR-TB are six to seven times higher among healthcare workers than in the general population (O’Donnell *et al.*, 2010). Furthermore, Wang *et al*. (2017) show how untrained bedside caregivers are more likely to be colonized or infected by DROs, due to a lack of hand hygiene education.

Women make up 70% of workers in the healthcare sector globally, particularly in nursing roles. For example, women make up 65% of nurses in Africa, 79% in South-East Asia, 81% in the Western Pacific and 86% in the Americas (Boniol *et al.*, 2019). In some regions such as sub-Saharan Africa, there is limited access to personal protective equipment (PPE) and low levels of IPC within the hospitals. Furthermore, even when PPE is available, ‘universal’ equipment is designed to fit larger users and therefore too large to fit smaller users, most of whom are women (Turner and Marshall, 2021). For example, gowns may be too long for women and drag on floors resulting in contamination. Gloves may not be available in small enough sizes. Visors, due to their length, may be dislodged by breasts, and ill-fitting facemasks and goggles may fail to seal properly on smaller female faces (Turner and Marshall, 2021). As such, wearing ‘universal size’ PPE poses higher risks for women due to gaps and inappropriate coverage. Furthermore, the men present in health care overwhelmingly tend to occupy higher positions, such as physician positions, than women. Men represent ∼72% of physicians in Africa (Boniol *et al.*, 2019). Physicians have less patient contact time compared with nurses, which lowers their risk of contracting infections from patients. Furthermore, as men tend to occupy higher and more powerful positions, women may have fewer tools to negotiate risk reduction interventions such as access to adequate PPE.

Lack of adequate IPC, coverage and PPE within hospitals heightens frontline healthcare workers’ vulnerability and exposure to infections and DROs present within their work environment, most of whom are women.

#### Animal husbandry

As alluded to earlier, proximity to animals, such as in the case of animal husbandry, compounded by poor WASH facilities, raises the risk of acquiring a DRO of zoonotic origin. Animal husbandry is the domain of both men and women, including children, depending on the type of animals reared. Gender determines how one is exposed to animals, shaping responsibilities around livestock in pastoralist and agro-pastoralist communities (Njuki and Sanginga, 2013; Barasa and Virhia, 2022). In smallholder systems, habitat overlaps between people and animals are common. Sometimes, young animals are kept inside or underneath people’s houses, particularly when they are ill (Barasa, 2019). Antimicrobials can be excreted by animals ‘virtually unchanged as the parent compound’, as they are frequently metabolized only partially in livestock and poultry (WHO, FAO & OIE, 2020). Frequent contact with medicated animals’ faeces reared within communities may therefore increase exposure to antimicrobials, which may lead to AMR.

Animal slaughter, commonly carried out without hygiene regulations in many LMICs, increases the risk of antibiotic resistance for entire communities, particularly consumers of unregulated urban abattoirs and butchers (Hrynick *et al.*, 2019). For example, the case of a processing plant in Limuru in Kenya highlights the extremely high prevalence of antibiotic resistance in *E. coli* from slaughtered broiler chickens (Adelaide *et al.*, 2008). In contexts such as pastoralist societies, the unhygienic handling of carcases and meat during the process of slaughtering animals is usually carried out by men (Barasa, 2019), which exposes them to higher risks of acquiring a DRO. Barasa highlights that adult pastoralist men in Tanzania spend less time in contact with livestock compared with women and herder boys, but their contact ‘often involves handling fresh carcasses and blood, inhaling aerosols and sprays when treating animals, assisting with animal abortions, and inspecting animal internal organs when slaughtering’ (2019, p. 112). ‘They also manage castration, vaccination, and slaughter, where they are likely to be exposed to sick animals and possibly to zoonoses’ (2019, p. 113). Women’s role in caring for sick animals not destined for slaughter in many livestock-keeping communities (Barasa, 2019) also increases their exposure to zoonotic pathogens. In this case, gendered roles in animal husbandry in pastoralist, agro-pastoralist communities or other low-resource settings may determine susceptibility to zoonoses, including potentially drug-resistant ones, ‘as certain individuals and groups may be more exposed to sick livestock than others’ (Barasa and Virhia, 2022, p. 2).

The handling of contaminated carcases is echoed in the handling of contaminated meat and the preparation of food, as highlighted later.

#### Food handling and preparation

One of the most common transmission routes for DROs is human contact with contaminated food (Godijk *et al.*, 2022). Food-borne diseases are likely to be higher in LMICs than in HICs because of the lack of surveillance of food-borne diseases, unhygienic food handling practices and consumption of food such as uninspected meat, fish and fresh produce (Uyttendaele *et al.*, 2016; Bisholo *et al.*, 2018). Furthermore, women and girls are largely in charge of household domestic labour, including food handling and preparation, which heightens their exposure to uncooked food, and potentially associated DROs.

Smoke inhalation while cooking is also associated with respiratory tract infections, which have the potential to become drug resistant, further heightening risks for women and girls. Pneumonia, for example, has a high potential to be antimicrobial resistant, and highly antimicrobial-resistant pneumonia is associated with higher mortality compared with other forms of pneumonia (Lakbar *et al.*, 2021). A study in South India has found that women had 2.89 higher odds of death compared with men, in cases of chronic obstructive pulmonary disease (often caused by exposure to noxious particles or gases), exacerbated by antimicrobial drug-resistant bacteria (Kaleem Ullah *et al.*, 2022). The WHO (2021b) estimated that 3.8 million people have died prematurely from indoor air pollution associated with inefficient cooking practices, of which 27% of deaths are from pneumonia. In rural Thailand, women and girls are largely in charge of cooking, usually with vegetable oil, using liquefied petroleum gas and without a ventilation hood (Juntarawijit and Juntarawijit, 2019). As such, fumes and smoke emanating from cooking increase the risk for women and girls of DRIs such as drug-resistant pneumonia and respiratory tract infections. Here, it is the very intersection of the gendered division of labour within the household and low-resource settings that increases the risks of exposure to DRIs for women and girls, through food handling and preparation.

#### Malnutrition

Epidemiological data have shown that malnutrition, which tends to be more widespread among women and children, increases the risk of morbidity and mortality from infections (Black *et al.*, 2013; Laxminarayan *et al.*, 2013; Unger *et al.*, 2019). Thus, malnutrition heightens one’s vulnerability to DRIs. Women and adolescent girls are more prone to malnutrition, particularly due to increased female nutritional needs after puberty, also associated with menstruation, pregnancy and lactation (Delisle, 2008). Globally, one-third of women of reproductive age are affected by anaemia (∼613 million women), reflecting high rates of malnutrition (UNSSCN, 2022). Malnutrition-induced anaemia increases women’s vulnerability to infections including DRIs.

Regarding children, maternal involvement in household decision-making along with maternal nutrition-related knowledge and educational status are independent predictors of child global acute malnutrition. As shown by a study in Ethiopia, three-fifths of mothers whose children suffered from acute malnutrition had poor nutrition-related knowledge (Girma and Alenko, 2020). In addition, cultural preferences for sons influence gendered child malnutrition, such as in India (Pillai and Ortiz-Rodriguez, 2015), where girls may receive less attention and breastfeeding time, as opposed to boys (Jayachandran and Kuziemko, 2011). This lack of food and subsequent undernourishment can exacerbate the risk of opportunistic infections and the likelihood of developing a DRI in these groups. Here, biological, sex-based differences in nutritional needs are disregarded by socio-cultural gender prioritization. The intersection of these cultural, gender and sex-based characteristics in low-resource settings increases the risk for women to develop an infection with the potential for drug resistance, through malnutrition.

#### Sexual behaviours

As alluded to so far, biological differences and differentiated vulnerability between males and females intersect with the differentiated risk of exposure of various at-risk groups, based on their behaviours or occupations. Here, we look at sexual behaviours, focusing on factors that may increase one’s risk of exposure to a DRI, through sex, gender, sexual orientation, occupation and geography.

In the case of potentially drug-resistant RTIs, such as *Neisseria gonorrhoea*, males and females have a differentiated experience of the infection. The male and female genital tract sensitivity to infection differs, and ‘transmission from males to females is more successful’ (Yakobi and Pooe, 2022). Furthermore, while males tend to experience symptoms when infected with gonorrhoea, females are usually asymptomatic and therefore may not be aware of the need to seek treatment (WHO, 2018; Yakobi and Pooe, 2022). When they do experience symptoms, these tend to be misdiagnosed as UTIs (Yakobi and Pooe, 2022), leading to incorrect antibiotics being prescribed, and failure to successfully treat the infection, which increases the risk of AMR. Furthermore, the lack of treatment in the long term may also cause damage to the reproductive system, as gonorrhoea can spread from the lower genital tract to the uterus or fallopian tubes and cause pelvic inflammatory disease (PID) (Jenning and Krywko, 2022). PID may lead to infertility in females, whereas this inflammation and its consequences are not likely for males (Jenning and Krywko, 2022). These biological sex-based differences interact with gender, occupation, sexual orientation and sexual behaviours, as outlined later, to increase or decrease one’s exposure and vulnerability to AMR.

Sexual orientation and associated sexual behaviours influence transmission patterns and vulnerability, as well as access to health care due to misconceptions or forms of discrimination. Sexual orientation may influence people’s exposure to pathogens, such as an increased risk of sexually transmitted infections (STIs) among men who have sex with men (MSM) who are a particularly high-burden group (Fairley *et al.*, 2017; Tsuboi *et al.*, 2021), due to high infection rates within this population (WHO, 2018). Gonorrhoea is of particular concern because rising rates will increase the probability of antimicrobial drug resistance (Fairley *et al.*, 2017). Yet, in the context of sexual orientation, stigma is a major deterrent to HSB for key populations such as MSM (Duby *et al.*, 2018). In sub-Saharan Africa, MSM face high rates of discrimination and stigma, including laws that criminalize homosexual relationships, sexual practices and identities, which creates barriers to prevention behaviours and accessing care services (Wanyenze *et al.*, 2016; Shangani *et al.*, 2018; Jerving, 2023). Many distance themselves from healthcare services due to the fear of negative interactions with healthcare providers, including the possibility of arrest or violence (Wanyenze *et al.*, 2016; Kushwaha *et al.*, 2017). There is a lack of protection for lesbian, gay, bi, trans, queer and other identities (LGBTQ+) individuals, impeding their access to STI treatment and sexual health services (Fay *et al.*, 2011), thus exacerbating vulnerability to DROs.

Some studies show that men are more likely to engage in risky sexual behaviours (RSBs) than women, which may put them at a higher risk of contracting drug-resistant gonorrhoea (DRG) (Nduna *et al.*, 2010; Kaggwa *et al.*, 2022). Studies in Africa showed that men have higher yearly gonococcal infection rates than women, with 100 new infections per 1000 men compared with 50 new infections per 1000 women (WHO, 2014). These significant gender-based differences are confirmed in that men tend to have higher numbers of sexual partners and more positive attitudes towards uncommitted sex, compared with women (Silva Júnior *et al.*, 2022). A study across 26 LMICs, including 21 sub-Saharan African countries, has shown that risk-taking sexual behaviour is ‘invariably associated with high educational attainment, urban residence and better wealth index’ regardless of the geographic location of the men who participated in the surveys (Berhan and Berhan, 2013). Other factors associated with increased RSBs, such as failure to use a condom or having transactional sex, may include depressive symptomatology in men (Nduna *et al.*, 2010). Furthermore, men purchasing sex are at particularly high risk of contracting STIs, including through heightened RSBs such as infrequent condom use during anal sex, substance use during sex and selling sex themselves (Rich *et al.*, 2020). All these could exacerbate the risk of developing STIs that are increasingly resistant to drugs, such as DRG.

Closely associated with the risks faced by men purchasing sex, the likelihood of acquiring a DRO is very high among sex workers, the majority of whom are women (WHO, 2012), who in many cases lack the power to negotiate safe sex with more powerful male clients due to structural inequities, financial pressure and gender power relations (Deering *et al.*, 2013). Sex workers are between 9 and 60 times more at risk of contracting STIs than the general population (Jeal and Salisbury, 2004). DRG is a prime example of this; ciprofloxacin-resistant gonorrhoea is common among sex workers in Kampala, Uganda (Vandepitte *et al.*, 2014), and penicillin-resistant *N. gonorrhoea* is more prevalent in sex workers in Ankara, Turkey (Zarakolu *et al.*, 2006). This high prevalence of AMR in this group may be explained through a combination of two factors; dense sexual network connectivity and antimicrobial drug exposure (Kenyon and Schwartz, 2018). On the one hand, sexual network connectivity sustains a high-equilibrium prevalence of gonorrhoea and increases the likelihood of reinfection. On the other hand, repeated antimicrobial drug exposure, such as in the case of screen-and-treat strategies for asymptomatic gonorrhoea, results in ‘selection pressure for reinfecting *N. gonorrhoea* strains to acquire antimicrobial-resistant genes’ (Kenyon and Schwartz, 2018, p. 1). In this case, the intersection of gender, socio-economic status and occupation places sex workers at a higher risk of contracting a DRI.

Sexual abuses also play a significant role in a differentiated infection rate between men and women. Women, including married women, are at a higher risk than men of being exposed to STIs through sexual violence, having a spouse with multiple partners (Mehta *et al.*, 2006), male resistance to condom use and women’s low ability to negotiate safe sex (Ali Abdulai *et al.*, 2017; Imo *et al.*, 2022). This may be explained partly by unequal norms about sexual morality and power (Go *et al.*, 2003; Bhattacharya, 2004; Cohen, 2004). A study across eight southern African countries highlighted men and women’s beliefs about gender violence: for example, 47% of men and 43% of women said that women do not have the right to refuse to have sex with their husbands or boyfriends (Andersson *et al.*, 2007). Furthermore, ∼35% of respondents considered that ‘forcing your partner to have sex is not rape’, and ∼24% considered that ‘forcing sex with someone you know is not rape’ either (Andersson *et al.*, 2007). A study based in North-West Ethiopia suggests a prevalence rate of intimate partner violence (IPV) of ∼49% among women of reproductive age (Getinet *et al.*, 2022). Another study in South-West Nigeria found an IPV prevalence of ∼38%, of which ∼80% of victims were women (Oseni *et al.*, 2022). Globally, the WHO (2021c) indicates that 30% of women have been subjected to physical and/or sexual IPV or non-partner sexual violence, in their lifetime. This increases women’s risk of contracting STIs, particularly gonorrhoea, one of the most transmitted STIs (Thomas *et al.*, 2002), which has the potential for drug resistance.

However, the lower prevalence of IPV among men ‘establishes the culture of silence; that is, IPV is more likely to be under-reported in men’ (Oseni *et al.*, 2022). This silence based on the stigma of IPV in men may therefore lead to a lack of treatment for infections and potentially drive the spread of DRIs.

The understanding of the various social stratifiers and behaviours outlined earlier (sex, gender, sexual orientation, sexual behaviours, geography, mental health, education, wealth, occupation and IPV, among others) captures the ‘clusters of disadvantage’ (Chambers, 2006), which impact sub-categories of the population with regard to their exposure and vulnerability to AMR, including, yet beyond, their gender.

### Gender and antimicrobial prescription, use and administration

HSBs can drive exposure to AMR, particularly in settings where access to prescription-only drugs is unregulated. These behaviours are often not linear and are made complex by the plurality of health systems as well as the inter- and intra-household power dynamics that influence healthcare decision-making in many low-resource settings. Antimicrobial prescription, administration and self-medication are keys to understanding people’s access to antimicrobials and rational or irrational use. Overall, education and knowledge play a crucial role in compliance with antibiotic treatment, lower expectations of antibiotic prescriptions and lower rates of self-medication (Bosley *et al.*, 2018). Women face more barriers to accessing health information than men as they are less likely to have the time to attend public health awareness events or free clinics on the safe use of antibiotics, especially when these are located away from home. Women generally receive less formal education than men across the globe (UNESCO, 2019), which has implications for antibiotic use for them and their children (Bosley *et al.*, 2018; Jones *et al.*, 2022).

Studies in LMICs have shown that the likelihood of prescribing or being prescribed antimicrobials is influenced by gender. Veterinary prescriptions for animal health seem to be influenced by the gender of a veterinarian. A study in Pakistan reveals that male veterinarians had higher odds of prescribing mass medication and use of antibiotics for disease than females, whereas improper disposal of expired antimicrobials was more common in female veterinarians (Saman *et al.*, 2023). Such study shows that, in spite of clinical guidelines, gender may influence the prescription of antibiotics in animal health.

In regard to receiving prescriptions in human health, women are 27% more likely than men to receive an antibiotic prescription in their lifetime (Aika *et al.*, 2022). The amount of antibiotics prescribed to women was 36% higher than for men in the 16–34 years of age group and 40% greater than in the 35–54 years of age group. These differences were particularly evident in cephalosporin and macrolide prescriptions in primary care (Schröder *et al.*, 2016). This gendered difference is attributed to the greater frequency of symptomatic infections in females than in males (particularly UTIs) (Schröder *et al.*, 2016). Studies suggest that men and women are more susceptible to having differentiated resistant pathogens based on what they have been prescribed throughout their lifetime (Aika *et al.*, 2022; Gözüküçük and Cakiroglu, 2023). In Turkey, when considering resistance rates for *Ureaplasma urealyticum*, ciprofloxacin resistance was found to be greater in women, while tetracycline resistance was found to be greater in men (Gözüküçük and Cakiroglu, 2023). The study suggests that women have more frequent UTIs, and using ciprofloxacin excessively increases the resistance rates in that group, while tetracycline/doxycycline is used extensively for urethritis in men (Gözüküçük and Cakiroglu, 2023). These examples show that gender influences exposure to antibiotics, which, in turn, increases the risk of exposure to AMR.

When antimicrobial prescription is neither sought nor accessible, self-medication can lead to an increased risk of AMR as the right dosage may not be observed and improper use is common. This includes treating viral infections with antibiotics, not buying the full course of treatment, interrupting treatment and sharing medicines, all of which can contribute to the emergence of resistant strains of bacteria. In the LMICs’ pharmaceutical sector, approximately two-thirds of antibiotics are used for self-medication (WHO, 2015). Self-medication is prevalent in some sub-Saharan countries such as Tanzania, Cameroon and Namibia, ranging from 50% to 60% in some regions (Simon and Kazaura, 2020), as well as in other LMICs such as India, with around three-quarters of antibiotics bought without prescriptions in some areas (Rousham *et al.*, 2023). Despite antibiotics being prescription-only medicines in these countries, they are still available and purchased in local drug stores and accessed through informal networks.

Attitudes towards drugs and lay aetiologies of illness (what should be treated at home, what is worth visiting a doctor for, who should be treated where and how) also influence HSBs that further compound the problem of AMR. For example, Barasa (2019) found that in pastoralist communities in Tanzania, lay attitudes drive herders to obtain penicillin for commonly occurring febrile illness, particularly in younger men, due to a belief that antibiotics ‘cure quickly’, enabling herders to return promptly to their work after a period of illness. Similar findings were recorded by Jones *et al.* (2022) in Nepal where men’s response to sickness is to seek ‘strong’ antibiotics as an initial treatment for any illness. While this irrational use of antibiotics may in fact be a driver of AMR, participants in the study describe men as seeking antibiotics to get better as fast as possible, in order to work. Medicines used for self-medication are often purchased without prescriptions from unlicensed local drug peddlers who sell unlabelled antibiotics in temporary nomadic shelters (Barasa, 2019), which increases the risk of AMR.

On the one hand, as shown in Uganda’s low-income regions, men tend to have higher rates of self-medication, due to greater purchasing power that enables them to obtain medication from the private sector, a more expensive option unavailable to women (Ocan *et al.*, 2014). Other studies have also shown that men are more likely to buy antibiotics without a prescription, compared with women (Jones *et al.*, 2022; Rousham *et al.*, 2023). Men also have higher rates of non-compliance with antibiotic treatment, which can expose them to higher rates of AMR. In Malaysia, 57% of people who did not comply with the full treatment were men (Fatokun, 2014). Non-compliance to treatment, including not purchasing the full course of antibiotics, can stem from reasons ranging from misinformation to financial barriers (Cambaco *et al.*, 2020). As such, increased education about antibiotic use is crucial to curb antimicrobial misuse, more prominent in men.

On the other hand, women as primary caregivers have a crucial impact on the medication of children and relatives they care for (Jones *et al.*, 2022). Across multiple studies, there is a noted tendency to reduce or stop the course of antibiotic treatment for children when symptoms are relieved or when they disappear, rather than complying with the full length of the prescription (Bosley *et al.*, 2018). The lack of women’s education and knowledge about antibiotics may have a strong effect on the misuse of antibiotics in children. For example, one study found that some breastfeeding mothers in Nigeria did not have an adequate understanding of AMR and AMU (Dadari, 2020). Maternal misuse of antibiotics while breastfeeding can contribute to the development of AMR, particularly in intestinal microbiota in a child, through breast milk transfer (Mathew, 2004). Furthermore, several studies found that mothers with a higher level of education, higher household income and private medical insurance were less likely to expect to receive antibiotics for their children (Bosley *et al.*, 2018), thus highlighting the importance of women’s education and knowledge about antibiotics for adequate usage of antibiotics in children.

Where the mothers have work commitments, however, they are more likely to medicate their children without seeing a doctor or without a prescription to avoid impinging on time spent earning an income for the family (Bosley *et al.*, 2018; Jones *et al.*, 2022). Therefore, the distribution of labour within the household has an impact on the likelihood of self-medication or medicating the children. These examples show how gender interacts with the household’s socio-economic status, decision-making power and education to shape the experience of health care and antibiotic treatment for all members of the family.

## Discussion

Throughout this paper, we used an intersectional gendered approach to highlight how sex and gender intersect with multiple social and biological stratifiers such as age, socio-economic circumstances, geography, occupation and others and how this results in differential lived experiences, exposure to DROs, vulnerability to DRIs and practices and attitudes towards AMU. As we have demonstrated, communities living in already under-resourced and remote settings have to negotiate a plethora of multiple and intersecting layers of disadvantage that lead to varying levels of health inequalities. These inequalities have implications for access to and use of antimicrobials and vulnerability to potential DRIs. Biologically, female and male bodies experience exposure and vulnerability to AMR in unique ways due to their anatomy and physiology, thus highlighting the importance of considering sex as an important variable in the study of AMR. Gendered norms and gendered roles in the domestic and public sphere further influence one’s exposure and vulnerability to AMR.

In the case of DRG, these social stratifiers intersect to heighten an individual’s vulnerability and exposure to the infection. For example, women’s subjection to sexual violence (Mehta *et al.*, 2006) is compounded by the asymptomatic female experience of gonorrhoea (WHO, 2018) and leads to increased exposure and vulnerability to DRG. This may also intersect with being a sex worker, a group that is at a much higher risk of contracting STIs, and with high drug-resistant strain prevalence, compared with the rest of the population (Jeal and Salisbury, 2004), including DRG (Zarakolu *et al.*, 2006; Vandepitte *et al.*, 2014). In parallel, men are generally more likely to engage in RSBs compared with women (Nduna *et al.*, 2010), which can heighten their exposure to DRG. In addition, their sexual orientation may further increase exposure to infection if have sexual relationships with men, as MSM are a high-burden group (Tsuboi *et al.*, 2021), and MSM are likely to face stigma when attempting to access sexual health services and STI treatment (Fay *et al.*, 2011). A good example of this is the recent legislation adopted in Uganda in March 2023, which further criminalizes homosexuality and may likely deter members of the LGBTQ+ community from seeking health services, for fear of facing a death sentence, or life imprisonment (Jerving, 2023). Incorporating an intersectional gender lens into health research and social epidemiology is therefore crucial to identifying and characterizing the most at-risk groups to AMR and hence ensuring interventions target those who are most in need of them. Without using such an intersectional gender approach, the nuances of individual experiences of illness, infection and most importantly DRIs are lost.

Another example that highlights the extent of such intersections is drug-resistant UTIs in the context of limited access to WASH facilities. Entire communities can face vulnerability to UTIs and drug-resistant UTIs can be in a state of flux, exacerbated by mutable and intersecting layers of marginalization, including those at the social and cultural interface. For instance, as discussed earlier, age intersects with gender and sex to increase younger women’s, pregnant women’s or older men’s vulnerability to UTIs (Akoachere *et al.*, 2012; Kumar, 2019; Yeta *et al.*, 2021; Lin *et al.*, 2022; Mohamed *et al.*, 2022). Prolonged hospitalization, or increased visits to health facilities, particularly those with limited access to WASH facilities, is a factor associated with an increased risk of contracting UTIs and drug-resistant UTIs (Ko *et al.*, 2008; Allegranzi *et al.*, 2011). This correlates with various life stages an individual encounters throughout their life, such as pregnancy, giving birth if women and old age, whereby patients spend more time in healthcare facilities. Age acts here as an intersecting factor that evolves throughout an individual’s life, alongside other factors such as geographical location, occupation and other contextual factors that may change through time.

In addition to vulnerability and exposure to DRIs, unlimited access to and inappropriate use of antimicrobials are further drivers of AMR. Some access barriers, which prevent people from seeking formal health care, and lead them to self-medicate or buy antibiotics at local drug peddlers, are easily visible, such as access to and distance from primary healthcare centres, stigma or discrimination, financial means or power and control over household financial resources, among others (Hossen and Westhues, 2011; Umubyeyi *et al.*, 2016; Minyihun and Tessema, 2020). Furthermore, HSBs can drive AMR more subtly as they manifest traditional attitudes towards drugs and illness aetiologies that influence narratives of lay people about healing and healthy bodies. These are entrenched, reinforced attitudes, which feed into HSBs, and influence knowledge exchange about medicine, within communities. These HSBs require a gender intersectional lens, to unpack the driving behaviours behind people’s health-seeking attitudes. For example, Jones’s research in Nepal (2022) uses a participatory approach and a gendered lens to understand drivers of AMU and misuse at the community level. By uncovering beliefs, perceptions and social norms related to care, illness and health, Jones provides insights into the motivations for seeking antibiotics and highlights how social norms differ between genders within community dynamics and may influence AMR-related behaviour.

An intersectional gender framework brings added value into the study of AMR, by uncovering some of the more subtle behaviours and attitudes that are lesser known, and yet they may contribute to the prevalence of DRIs or to the drivers of AMR. Rather than focusing on where the burden of AMR is the highest, we argue that such a framework is key to understanding specific people who may bear the greatest brunt of the burden of AMR in community settings.

Literature published on AMR tends to take the epidemiological data out of context and only talk about AMR in ‘drugs and bugs’ terms. The global burden of bacterial AMR data published in *The Lancet* (Murray *et al.*, 2022), which is the best estimate today of the AMR burden globally, for instance, does not yet consider how the burden of AMR in terms of morbidity, mortality or economic costs varies by gender or any other socio-economic characteristics. There is, therefore, a need for disaggregated AMR surveillance and burden data by sex, gender, ethnicity, geography, cultural and religious practices and socio-economic status to better understand how AMR affects and impacts various segments of the population.

While sex is an accessible metric to collect and use in AMR research, other metrics such as gender (particularly if non-binary or trans) or sexual orientation may be difficult to collect due to discrimination, and unsafe for a patient to disclose, when accessing health care. Other data such as cultural practices may also not be possible to represent on an epidemiological level. Nevertheless, this is where anthropological approaches may come into the picture to allow a contextual approach of AMR, at the community level.

This paper has shown how gendered lived experiences of AMR are different based on where and how one lives. We argue that ignoring how gender and other social stratifiers influence the burden of AMR masks people’s lived experience of health care and exposure to AMR. Studying AMR without considering how gendered intersections shape people’s lives lacks the precision to truly understand how to tackle the burden and spread of this silent and overlooked pandemic (Laxminarayan, 2022).

Health systems are not gender neutral, and they are not always accessible, affordable and acceptable to all groups within the population (Theobald *et al.*, 2017). Gender is ‘a key determinant of health in any given society’ (WHO, 2018), which influences people’s experience of and access to health care. Sex-disaggregated data are a key element in flagging female–male differences or similarities, which should act as a trigger for further gender and intersectional analyses to better understand how social inequalities lead to different experiences within health systems (Morgan *et al.*, 2016).

## Conclusion

Our analysis of the literature sourced through our review has demonstrated the need for further research using an intersectional gender lens to unpack how sex, gender and other social stratifies interact in different contexts to drive both the burden and the spread of AMR. In order for such research to be possible, national AMR surveillance systems as well as AMU monitoring systems (such as point prevalence surveys) and AMR burden estimations need to be designed so that they will be able to collect and collate data disaggregated by sex, gender, age, education, occupation, ethnicity, place of residence and other relevant social stratifiers. In addition to quantitative data, there is also a need for additional research taking a social science and anthropological approach to uncover the social underpinnings of AMR drivers.

Building on both quantitative and qualitative evidence, we suggest that health researchers working on interventions to avert the burden of AMR should consider applying an intersectionality approach in order to better understand exposure to, vulnerability to and drivers of AMR for different people in different contexts. Similar approaches including One Health that foster interdisciplinarity should also be considered, including operational research on transmission routes of AMR between humans and between humans, animals and the environment within specific communities.

This would have several benefits. First, the evidence generated could help practitioners (both in the human and animal health side) to improve treatment of infections within their communities, thereby contributing to lessening the burden of infections including DRIs and decreasing the need for using antimicrobials overall. Second, this analysis could and should be used to better tailor measures to prevent infection (such as better WASH and waste management in the human, animal and environmental sectors and better IPC). Third, this evidence could inform initiatives to lower people’s vulnerability to becoming ill (such as campaigns or programmes to strengthen access to vaccination or improve nutrition for communities that are traditionally missed). Finally, this could also drive interventions to improve access to quality diagnostics and quality-assured treatment for all (such as interventions to lower or remove out-of-pocket fees for accessing health care, measures to transform social norms that shape the distribution of knowledge and decision-making power regarding health choices within households, social and behavioural change communication campaigns to promote rational use of antimicrobials and AMR stewardship programmes).

Integrating an evidence-based intersectional gender lens in all policies and plans to tackle AMR, starting with national AMR action plans, will not only increase their chances to be effective at promoting rational use of antimicrobials for all but also lessen the burden of AMR (especially for those who are disproportionately affected). It would also help tackle the spread of AMR (ensuring that existing antimicrobials will still be effective in the future when needed) and contribute to global-level goals such as the drive towards Universal Health Coverage, the Immunization 2030 agenda and ultimately the sustainable development goals with their promise to leave no one behind.

## Reflexivity statement

The authors are a diverse group of women of different ages, seniority, nationalities and ethnicities, thus reflecting our commitment to gender, diversity and inclusion and also mirroring this paper’s intersectional approach that aims to consider the variety of personal experiences (and their health implications) that arise from multiple social stratifiers. J.M.C.G., an international development consultant with expertise in health and gender and PhD candidate in social anthropology, led the analysis for relevant publications and writing of the paper, with inputs from G.T.T., a seasoned international development consultant with extensive experience in AMR, other pandemics and gender, who designed and oversaw the study and with V.B.’s critical revision as an expert in gender and development, bringing in perspectives and lived experiences from the global south where her research is based.

The gendered composition of our group may have made us more biased towards the experiences of women. It is indeed important to be sensitive to such gendered experiences to appropriately highlight the vulnerabilities of women and the discrimination they may face when accessing health services, particularly in settings where, as we will show in the paper, gender inequalities shape this access. Nevertheless, we have also shown how men may also be at increased risk of DRIs (e.g. MSM, men working in animal slaughter and the prevalence of self-medication in men) and have applied a non-binary definition to gender, despite the gap in the literature on non-binary individuals and AMR.

## Appendix

**Table A1.** Search terms used, by thematic area

AMR-related search	Gender-related searches	Specific thematic searches
‘antimicrobial resistance (AMR)’ ‘antibiotic resistance’ ‘drug-resistant infection (DRI)’ ‘drug-resistant organism (DRO)’ ‘multidrug-resistant organism’	‘gender’ ‘women’ ‘men’ ‘sex’ ‘male’ ‘female’	‘pregnancy’ ‘childbirth’ ‘UTI’ ‘menstrual hygiene management’ ‘UTI’ ‘WASH’ ‘water provision’ ‘healthcare workers’ ‘animal rearing’ ‘animal slaughter’ ‘cooking’ ‘nutrition’ ‘malnourishment’ ‘sex work’ ‘men who have sex with men (MSM)’ ‘sexual behaviours’ ‘sexual and gender-based violence (SGBV)’ ‘risky sexual behaviours’ ‘prescription of antibiotics’ ‘self-medication’ ‘compliance to treatment’
